# Phlegmons of the Upper Respiratory Tract

**Published:** 1913-09

**Authors:** John O. Roe

**Affiliations:** Rochester


### Phlegmons of the Upper Respiratory Tract.

The phlegmon is simply another name for inflammation, although clinically it is regarded as an extraordinarily intense inflammation and looked upon as being of more or less malignant character. We may have an inflammation of this character in any part of the body, the intensity of which may depend upon the virulence of the infection and the resistance or lack of resistance of the patient, or of the tissues, to such infection. Owing to the difficult breathing or choking which such inflammation causes in the upper air passages, it has received the name of angina. Inflammations of a severe grade, termed phlegmons, may involve any portion of the upper respiratory tract. We almost invariably find a phlegmon attacking one whose general condition has become weakened. The treatment of these infections requires no special law unto itself, and must be dealt with according to the location and associated complications.—Dr. John O. Roe, Rochester.

